# True isolated deep femoral artery aneurysm associated with peripheral artery disease: case report

**DOI:** 10.1590/1677-5449.200220

**Published:** 2021-06-16

**Authors:** Vinicius Tadeu Ramos da Silva Grillo, Rodrigo Gibin Jaldin, Nathália Dias Sertório, Matheus Bertanha, Marcone Lima Sobreira, Ricardo de Alvarenga Yoshida, Winston Bonetti Yoshida

**Affiliations:** 1 Universidade Estadual Paulista “Júlio de Mesquita Filho” – UNESP, Hospital das Clínicas da Faculdade de Medicina de Botucatu – HC-FMB, Serviço de Cirurgia Vascular e Endovascular, Botucatu, SP, Brasil.

**Keywords:** femoral artery, aneurysm, peripheral arterial disease, vascular surgical procedures

## Abstract

True deep femoral artery aneurysms are extremely rare, accounting for about 0.5% of all peripheral aneurysms. In this report, we describe a 79-year-old male patient with a history of prior abdominal aortic aneurysm surgery via a conventional approach who was admitted to the vascular surgery service at the Hospital das Clínicas with intermittent claudication of the lower limbs. Arterial color-Doppler ultrasonography of the right lower limb was performed, revealing peripheral arterial disease of the femoral--popliteal and infrapatellar segments. Computed tomography angiography identified aortoiliac and bifurcated graft occlusion from the infrarenal segment of the aorta, in addition to a deep femoral artery aneurysm with diameters of 3.7 cm x 3.5 cm and length of 7 cm. Resection of the aneurysm was followed by revascularization of the deep femoral artery by interposition of a Dacron® graft and reimplantation of the superficial femoral artery into the graft. In cases of deep femoral artery aneurysms with concomitant peripheral arterial disease, it is important to ensure revascularization and adequate perfusion of the lower limb.

## INTRODUCTION

Aneurysms of the deep femoral artery (DFA) are defined as those that do not continue to an aneurysm of the common femoral artery (CFA). True DFA aneurysms are extremely rare, accounting for around 0.5% of all peripheral aneurysms and 1-2.6% of femoral aneurysms.^1^^-^5 In the majority of cases, etiology is atherosclerotic and they are more common in men (92%) with a mean age of approximately 70 years.^6^^-^8

There are no precise criteria in the current literature for defining surgical indications or the best treatment techniques for aneurysms of the DFA, which is a result of their rarity and the scarcity of studies of their natural history.^4^^,^^5^^,^^7^^,^^9^ In this study, we describe an option for surgical treatment in a patient with an isolated DFA aneurysm with concomitant peripheral arterial disease (PAD). The Research Ethics Committee approved this study (decision number 4.699.360).

## CASE REPORT

The patient was a 79-year-old white male with Japanese ancestry who was seen at the vascular surgery service of the Hospital das Clínicas complaining of pain in the right hip and lower limb intermittent claudication limiting him to 100 meters, with no pain at rest or trophic lesions, but which had worsened over the previous 4 months, increasing in intensity. He had a history of systemic arterial hypertension, was an ex-smoker (five pack-years), having quit more than 50 years previously, and had been treated with aortoiliac interposition of a bifurcated Dacron® graft to repair an infrarenal aortic aneurysm at a different service 16 years before. The graft had become occluded 5 years before the current presentation.

On physical examination, he was in good general health, with an abdominal scar from the previous laparotomy. A pulsating bulge with fibroelastic consistency was palpable in the right inguinal region. Femoral pulses were weak bilaterally (2+/4+) and popliteal and distal pulses were absent. Ankle-brachial indices (ABI) were calculated bilaterally. On the right, Doppler ultrasound did not detect flow in the anterior tibial artery and the posterior tibial and fibular arteries had ABIs of 0.42 and 0.21 respectively. On the left, ABIs for the anterior tibial, posterior tibial, and fibular arteries were 0.47, 0.63, and 0.42, respectively. There were no trophic ulcers.

**Figure 1:**
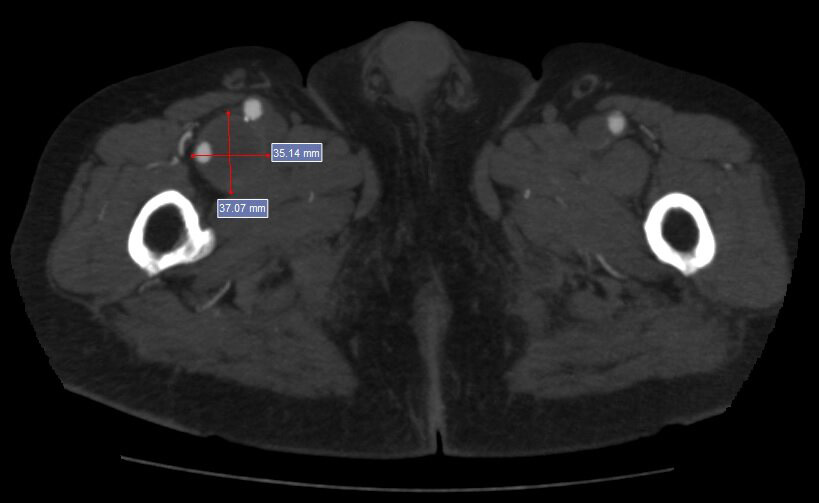
Axial image from computed tomography angiography (CTA) of the proximal thigh, showing an aneurysmal dilatation of the deep femoral artery (DFA) with diameters of 3.7 cm (anteroposterior) and 3.5 cm (lateral) and intraluminal thrombi.

**Figure 2:**
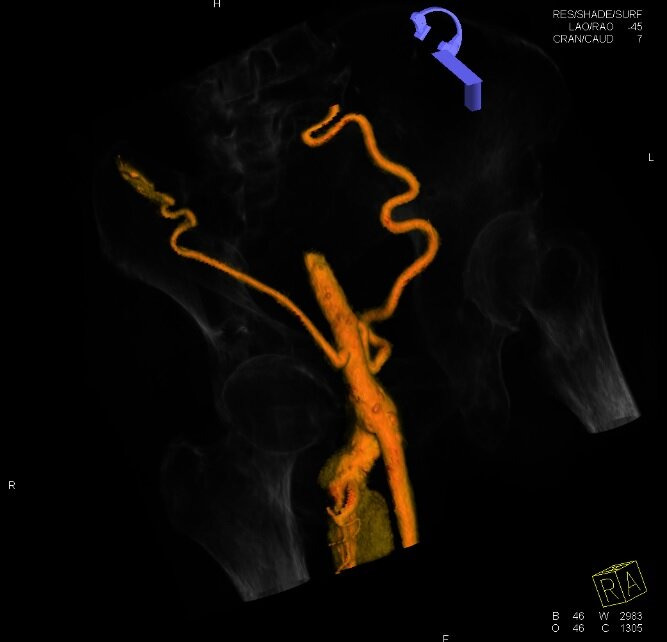
Three-dimensional reconstruction of computed tomography angiography (CTA) with bony landmarks, showing refilling of the common femoral artery (CFA) by collaterals and the aneurysmal dilatation of the deep femoral artery (DFA), with no involvement of the CFA or of the superficial femoral artery (SFA).

A supplementary examination was performed using color Doppler ultrasonography (CDU) of the arteries of the right lower limb, revealing stenosis and segmental subocclusion of infrapatellar arteries. The patient was sent for computed tomography angiography (CTA) of the abdomen and pelvis, which identified aortoiliac occlusion (within the Dacron® graft) starting in the infrarenal segment of the aorta. The CFAs were patent because of refilling via large caliber collateral circulation originating in the inferior epigastric arteries and the internal thoracic arteries. Additionally, a DFA aneurysm was found with diameters of 3.7 and 3.5 cm (anteroposterior and lateral, respectively), length of 7 cm (Figure 1), and intraluminal thrombi. Image reconstruction was employed to enable analysis of the aneurysm’s anatomic relationships (Figure 2).

Since surgery was needed, a preoperative risk assessment was performed, classifying the patient as at moderate risk of cardiovascular complications.^10^ Since technical and tactical difficulties were expected, endovascular techniques were ruled out because of the postoperative history of aortoiliac revascularization with occlusion of the graft and lower limb PAD.

**Figure 3:**
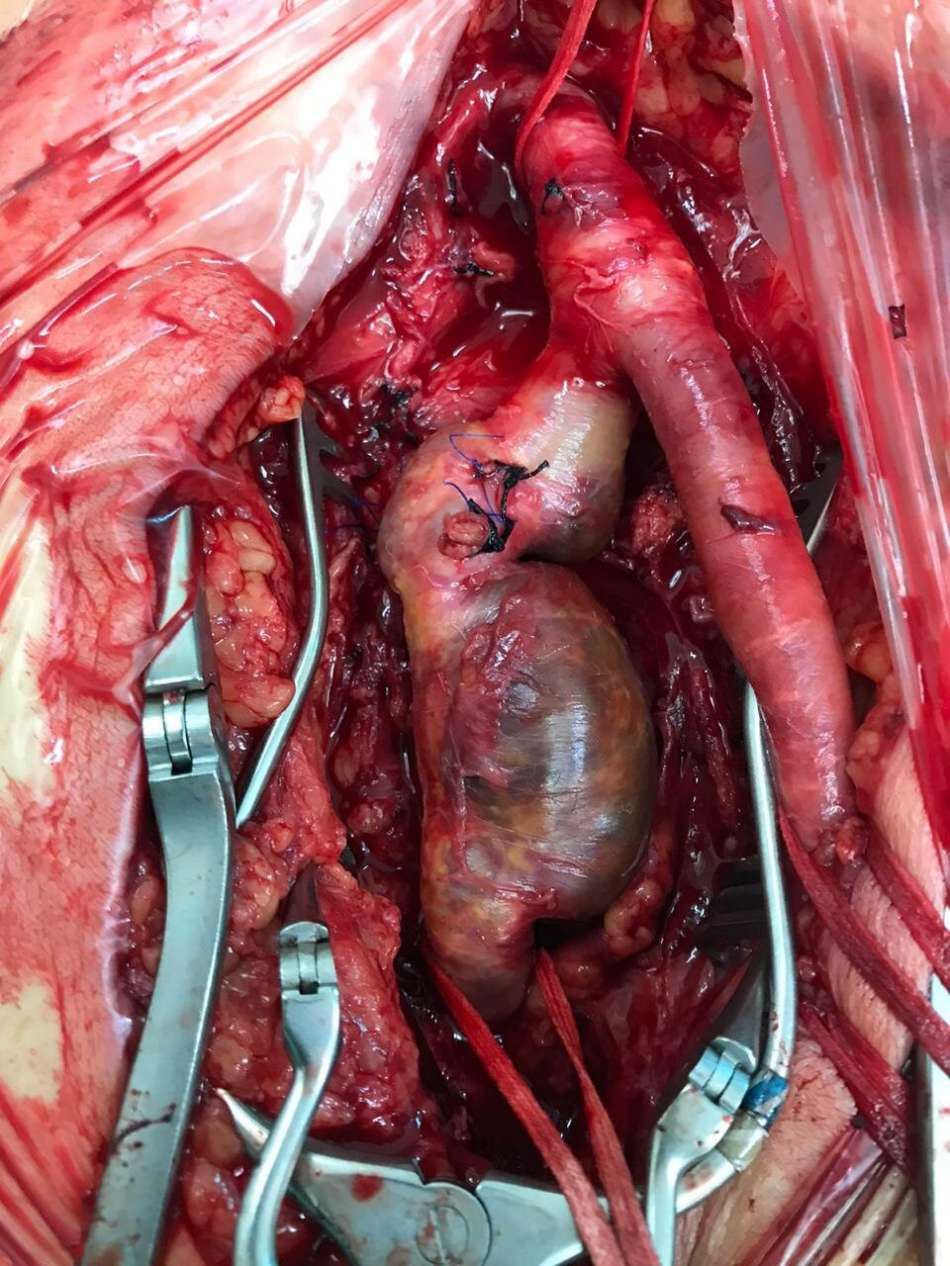
Result of surgical dissection, after proximal repair of the common femoral artery (CFA) and distal repair of the superficial femoral artery (SFA) and deep femoral artery (DFA) beyond the dilation. Note the ligature of branches from the DFA aneurysm.

**Figure 4:**
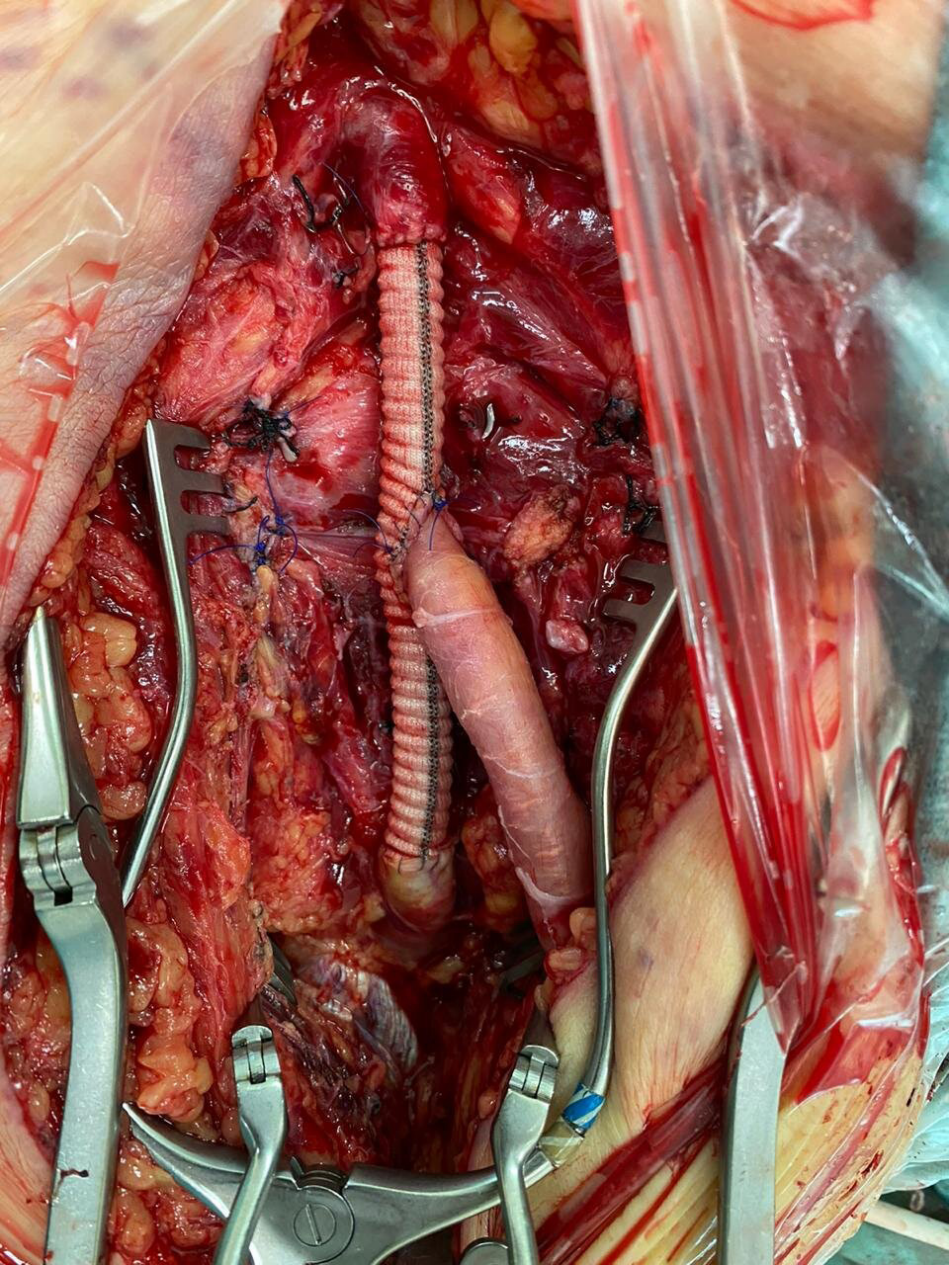
Result of revascularization, with interposition of Dacron® graft, proximal anastomosis with the common femoral artery (CFA), and distal anastomosis with the deep femoral artery (DFA), combined with reimplantation of the superficial femoral artery (SFA) into the graft.

**Figure 5:**
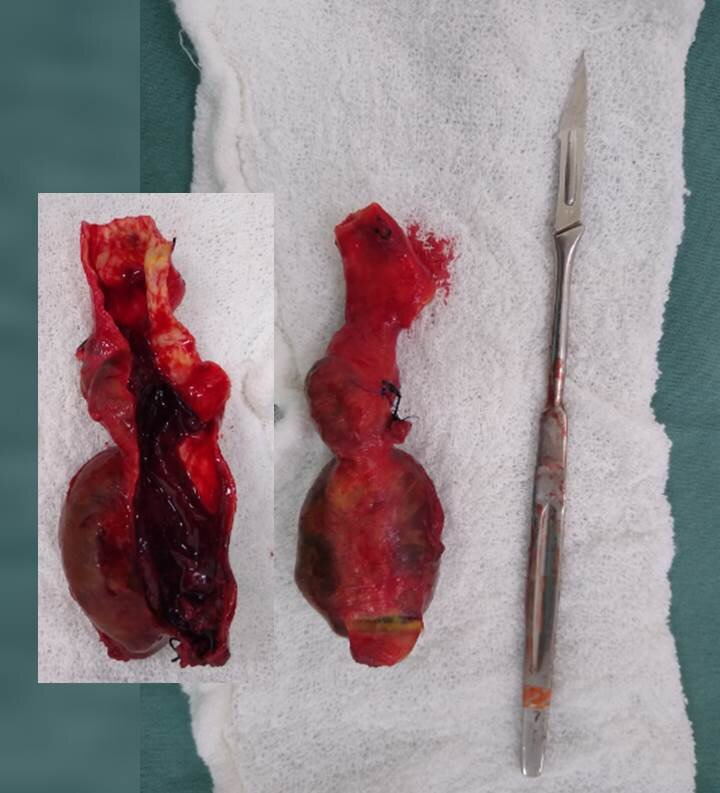
Surgical specimen. Morphology of the aneurysm and its relationship to the dimensions of a number 7 scalpel handle and number 11 blade. Insert: morphology of the aneurysm after opening, showing intraluminal thrombi.

**Figure 6:**
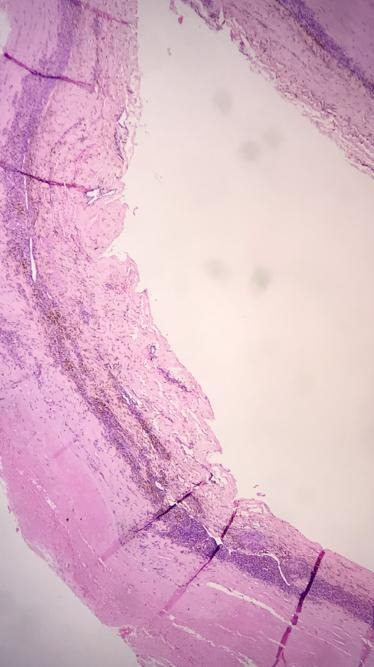
Histopathological analysis. H&E staining: x400 magnification. Artery with dilated lumen, degenerate tunica media and adventitia with chronic inflammation.

**Figure 7:**
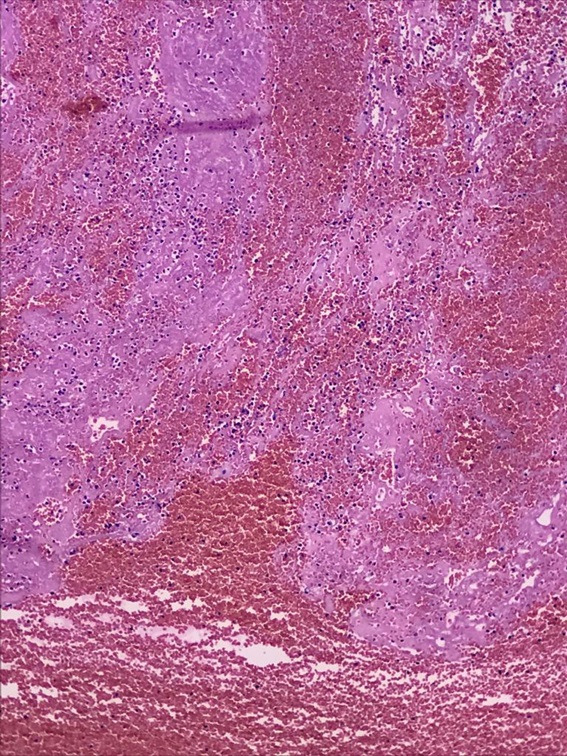
Histopathological analysis. H&E staining: x400 magnification. Acute fibrin-hematic thrombus in the process of organization.

Surgical access was achieved via a large vertical groin incision, slightly lateral of the conventional approach. The CFA and superficial femoral artery (SFA) were identified and isolated, both of which were ectatic, followed by the DFA, which exhibited an aneurysm starting distal of its origin at the bifurcation and following a posterior-inferior course for approximately 7 cm before the caliber returned to normal (Figure 3). Resection of the aneurysm and revascularization of the DFA were performed. Techniques such as embolization or ligature were ruled out because of the coexisting diffuse atheromatosis of the SFA and occluded arteries of the leg, since the DFA was considered an important source of collateral circulation to perfuse the limb. After safely isolating the proximal and distal arterial segments and positioning the DeBakey atraumatic clamps, the aneurysmal segment was resected. The DFA was revascularized first, to reduce the duration of limb ischemia, since the coexisting PAD meant that the DFA was responsible for distal perfusion. Next, an 8 mm Dacron® graft was interposed end-to-end from the CFA to the healthy distal neck of the DFA and then the SFA was sectioned and reimplanted into the prosthesis, ensuring the path with the best anatomic fit (Figure 4). At the end of the procedure, pulses were palpable before and after the anastomoses with no asymmetry or thrill. Since the patient was neither claudicant nor ischemic, the decision was taken not to perform conventional or endovascular distal revascularization, since to do so could have increased the morbidity and mortality of the procedure. A vacuum drain was fitted into the subcutaneous tissue because of the large dead-space caused by the size of the aneurysm. The surgical specimen (Figure 5) was sent for pathological analysis, which detected probable atherosclerotic origin, with calcified plaque and fibrin-hematic thrombus in the lumen (Figures 6 and 7). The patient progressed well, the drain was removed on the second postoperative day (POD), and he was discharged from hospital on the fifth POD. At 14 and 30-day outpatient follow-ups, the surgical wound was fully healed over and the patient had no complaints of pain or vascular or neurological disorders. Physical examination found that the palpable femoral pulses were maintained bilaterally (2+/4+) and popliteal and distal pulses were still absent. The extremity of his right lower limb was warm and his capillary refill time was less than 3 seconds.

## DISCUSSION

A systematic review of articles published in English identified 46 reports of true isolated DFA aneurysms.^6^ Another review, from the previous year, identified a series of just over 50 cases reported.^7^ Recently, approximately 140 cases of DFA aneurysms were described in the literature,^11^ showing that, although the numbers are increasing, they nevertheless illustrate the rarity of this clinical entity.

Generally, aneurysms of the femoral arteries are found in combination with aneurysms of other arteries, such as the aorta and lower limb arteries, and they are bilateral in 5% of cases.^1^^,^^4^^-^8 In 50% of cases, there is an association with PAD in the femoropopliteal area.^4^ False aneurysms and pseudoaneurysms are more common in the literature, primarily posttraumatic and iatrogenic cases.^6^^,^^12^ It is considered that the low incidence is because of containment by the adjacent muscular structures, in particular the adductor magnus muscle, surrounding and protecting the artery.^3^^,^^5^^-^8^,^^11^

The difficulty of clinical diagnosis, particularly when the aneurysm is small, is reflected in the frequency of asymptomatic cases or cases with nonspecific lower limb symptoms, such as pain, edema, and neurological symptoms.^3^^-^7^,^^9^^,^^11^ The most common finding is a pulsating mass with or without pain, but these aneurysms can also be detected as incidental findings of supplementary examinations.^3^^,^^6^ There is also a report of differential diagnosis for incarcerated inguinal hernia, primarily in the elderly, which can have disastrous consequences if not diagnosed before the operation.^2^

There is a high risk of complications with DFA aneurysms, related to the difficulty in diagnosing them and the consequent longer period for which the disease remains untreated, which can result in the patient’s initial presentation being thrombosis, distal embolization, and rupture.^4^^,^^9^ Rupture is the most common complication and the risk varies from 15 to 55%, with a direct relationship between aneurysm size and risk of rupture, while DFA aneurysms generally have a larger diameter than those of the SFA.^3^^,^^6^^,^^7^^,^^13^ The difficulties hampering reconstruction caused by distorted anatomy contribute to the high amputation rates when these aneurysms rupture.^4^^,^^6^

Several different surgical approaches are described in the literature,^11^ including ligature and resection of the aneurysm in isolation^6^^-^8^,^^11^ or combined with revascularization^4^^,^^9^ using synthetic or autologous grafts, and also endovascular procedures involving implantation of covered stents^1^^,^^13^ and embolization, although there is still a lack of data on the long-term patency of covered stents in this area.^1^ Limitations restricting use of endovascular treatment can include extension of the disease to involve the CFA or absence of a large enough proximal landing zone to enable sealing. Ligature is considered safe, particularly when distal pulses are present and the femoral-popliteal segment is patent, but every effort should be made to achieve revascularization when the distal pulse is absent.^6^^,^^7^^,^^9^^,^^11^ The DFA plays an important role in collateral circulation to the lower limbs in patients with PAD, which is progressive over the years.^4^

In order to confirm the diagnosis with certainty, CDU, CTA, and angiography are extremely useful. Multi-detector CTA or angiography is especially recommended because they will reveal other aneurysms and stenotic or occlusive arterial lesions.^3^^,^^7^ It is also necessary to take account of the patient’s anatomy and changes to it, since there are descriptions of anatomic variants in this area that could result in disaster during procedures if they are not identified in advance.^14^

## CONCLUSIONS

In this case, the patient had an aneurysm of the DFA and PAD and, as a result, it was necessary to perform revascularization to maintain collateral circulation and sufficient perfusion of the lower limb. The patient was offered a safe and effective surgical therapeutic option to treat the disease, without short-term postoperative complications. It should therefore be considered that conventional surgery has an important role to play in treatment of aneurysms involving the DFA.
