# Supplementary material for: True isolated deep femoral artery aneurysm associated with peripheral artery disease: case report
Source: J Vasc Bras. 2021 Jun 16;20:e20200220. doi: 10.1590/1677-5449.200220 (PMC8218824; doi:10.1590/1677-5449.200220)
Supplement: Translation

# Translation: Aneurisma verdadeiro isolado de artéria femoral profunda associado a doença arterial periférica: relato de caso

**Authors:** Vinicius Tadeu Ramos da Silva Grillo, Rodrigo Gibin Jaldin, Nathália Dias Sertório, Matheus Bertanha, Marcone Lima Sobreira, Ricardo de Alvarenga Yoshida, Winston Bonetti Yoshida

**Affiliations:** 1 Universidade Estadual Paulista “Júlio de Mesquita Filho” – UNESP, Hospital das Clínicas da Faculdade de Medicina de Botucatu – HC-FMB, Serviço de Cirurgia Vascular e Endovascular, Botucatu, SP, Brasil.

**Keywords:** artéria femoral, aneurisma, doença arterial periférica, procedimentos cirúrgicos vasculares

## Abstract

Os aneurismas verdadeiros de artéria femoral profunda são extremamente raros, representando cerca de 0,5% do total dos aneurismas periféricos. Neste relato, descrevemos um paciente de 79 anos de idade, sexo masculino, com histórico de abordagem cirúrgica prévia convencional devido a aneurisma de aorta abdominal, que deu entrada no Serviço de Cirurgia Vascular do Hospital das Clínicas com quadro de claudicação intermitente de membros inferiores. Foi realizado eco-Doppler colorido arterial do membro inferior direito, que revelou doença arterial periférica femoro-poplítea e infrapatelar. A angiotomografia computadorizada identificou oclusão aortoilíaca e do enxerto bifurcado desde o segmento infrarrenal da aorta, além de aneurisma de artéria femoral profunda de 3,7 x 3,5 cm de diâmetro com 7 cm de extensão. Procedeu-se com a ressecção do aneurisma e revascularização da artéria femoral profunda por interposição de prótese de Dacron® e reimplante de artéria femoral superficial na prótese. Portanto, nos casos de aneurisma de artéria femoral profunda concomitante a doença arterial periférica, deve-se atentar para revascularização e perfusão adequada do membro inferior.

## INTRODUÇÃO

Considera-se aneurisma isolado da artéria femoral profunda (AFP) aquele que não possui continuidade com o aneurisma da artéria femoral comum (AFC). Os aneurismas verdadeiros de AFP são extremamente raros, representando cerca de 0,5% do total dos aneurismas periféricos e 1-2,6% dos aneurismas femorais^1^^-^5. A etiologia é, em sua maioria, aterosclerótica e são mais comuns em homens (92%), com idade média de aproximadamente 70 anos^6^^-^8.

Diante de sua raridade e da escassez de estudos da evolução natural dos aneurismas de AFP, não há, na literatura atual, critérios precisos para sua indicação cirúrgica nem qual seria a melhor técnica de tratamento^4^^,^^5^^,^^7^^,^^9^. Neste estudo, relatamos uma opção de tratamento cirúrgico em paciente portador de aneurisma isolado de AFP associado à doença arterial periférica (DAP). O protocolo foi aprovado pelo Comitê de Ética de nossa instituição (parecer número 4.699.360).

## RELATO DE CASO

Paciente de 79 anos de idade, sexo masculino, branco, ascendência japonesa, avaliado no Serviço de Cirurgia Vascular do Hospital das Clínicas com queixa de dor em quadril direito e claudicação intermitente de membros inferiores para 100 metros, sem dor em repouso ou lesão trófica, que evoluiu com piora da intensidade há aproximadamente 4 meses. Histórico de hipertensão arterial sistêmica, ex-tabagista (cinco maços-ano), tendo cessado há mais de 50 anos, foi submetido à cirurgia de interposição arterial aortoilíaca com prótese Dacron® bifurcada por aneurisma de aorta infrarrenal em outro serviço há 16 anos, que se apresentava com oclusão há cinco anos.

Ao exame físico, apresentava-se em bom estado geral, com cicatriz abdominal de laparotomia prévia. Na região inguinal direita, palpava-se abaulamento de consistência fibroelástica e pulsátil. Os pulsos femorais apresentavam-se diminuídos bilateralmente (2+/4+), e os pulsos poplíteos e distais estavam ausentes. Foram mensurados os índices tornozelo-braquial (ITB) bilateralmente: à direita, a artéria tibial anterior não tinha fluxo identificável ao Doppler ultrassom, e as artérias tibial posterior e fibular estavam com o ITB de 0,42 e 0,21, respectivamente. À esquerda, registrou-se ITB de artérias tibial anterior, tibial posterior e fibular de 0,47, 0,63 e 0,42, respectivamente. Não havia presença de lesões tróficas.

Realizou-se ainda estudo complementar por eco-Doppler colorido (EDC) arterial de membro inferior direito, que identificou estenoses e suboclusões segmentares das artérias infrapatelares. O paciente foi submetido à angiotomografia computadorizada (ATC) de abdômen e pelve, sendo identificada oclusão aortoilíaca (intraprótese de Dacron®) desde o segmento infrarrenal da aorta. As AFCs apresentavam-se pérvias por reenchimento por circulação colateral de calibre considerável, oriunda das artérias epigástricas inferiores e torácicas internas. Identificou-se aneurisma de AFP de 3,7 x 3,5 cm de diâmetro (anteroposterior e laterolateral, respectivamente) com 7 cm de extensão (Figura 1) e com trombos em seu interior. Foi reconstruída a imagem para avaliação das relações anatômicas do aneurisma (Figura 2).

**Figura 1:** Angiotomografia computadorizada (ATC) em corte axial da coxa proximal, com identificação da dilatação aneurismática da artéria femoral profunda (AFP) com dimensões de 3,7 cm (anteroposterior) e 3,5 cm (laterolateral), com trombos em seu interior.

**Figura 2:** Reconstrução tridimensional de angiotomografia computadorizada (ATC) com referências ósseas, observando-se reenchimento de artéria femoral comum (AFC) por colaterais e dilatação aneurismática da artéria femoral profunda (AFP), sem envolvimento da AFC e artéria femoral superficial (AFS).

Indicada a abordagem cirúrgica, realizou-se avaliação de risco pré-operatório, que classificou o paciente como risco moderado risco para complicações cardiovasculares^10^. Antevendo a dificuldade relacionada à técnica e à tática, foi descartada a abordagem endovascular devido ao histórico do pós-operatório de revascularização aortoilíaca com oclusão da prótese associado a DAP dos membros inferiores.

O acesso cirúrgico deu-se por inguinotomia longitudinal ampla, discretamente lateralizado em relação à abordagem convencional, com identificação e isolamento da AFC e artéria femoral superficial (AFS), que se encontravam ectasiadas, e da AFP, a qual apresentava aneurisma que iniciava após a sua origem na bifurcação, seguia trajeto póstero-inferior por aproximadamente 7 cm e normalização do calibre após (Figura 3). Foram realizadas ressecção do aneurisma e revascularização da AFP. Não foram consideradas técnicas como embolização ou ligadura, dada a coexistência de ateromatose difusa da AFS e oclusão de artérias da perna, considerando a AFP como importante fonte de circulação colateral para perfusão do membro. Após isolamento seguro dos segmentos arteriais proximal e distal e posicionamento de clampes atraumáticos tipo DeBakey, procedeu-se a ressecção do trajeto aneurismático. Optou-se, inicialmente, por revascularização da AFP para diminuir o tempo de isquemia do membro, visto que, pela associação com DAP, a AFP era a responsável pela perfusão distal. Na sequência, procedeu-se com a interposição término-terminal de prótese de Dacron® 8 mm entre AFC e colo distal saudável de AFP e posterior secção e reimplante da AFS na prótese, garantindo trajeto de melhor caimento anatômico (Figura 4). Terminado o procedimento, foram palpados pulsos pré e pós-anastomoses, sem a presença de assimetria ou frêmito. Como o paciente não era claudicante nem estava isquêmico, optou-se por não realizar a revascularização distal, convencional ou endovascular, pois poderia aumentar a morbimortalidade do procedimento. Frente à grande área de espaço-morto provocado pelas dimensões do aneurisma, optou-se por alocação de dreno a vácuo no tecido celular subcutâneo. A peça cirúrgica (Figura 5) foi enviada para análise anatomopatológica, que revelou provável origem aterosclerótica, com placa calcificada e trombo fibrinoleucocitário no lúmen (Figuras 6 e 7). O paciente apresentou boa evolução, o dreno foi retirado no segundo dia de pós-operatório (DPO), recebendo alta hospitalar no 5º DPO. Nos retornos ambulatoriais de 14 e 30 dias, o paciente apresentava cicatrização total da ferida operatória, sem queixas álgicas, vasculares ou neurológicas. No exame físico, mantinha os pulsos femorais palpáveis bilateralmente (2+/4+), e os pulsos poplíteos e distais estavam ausentes. Apresentava-se com extremidade do membro inferior direito quente e tempo de enchimento capilar menor do que 3 segundos.

**Figura 3:** Resultado da dissecção cirúrgica, após reparo proximal da artéria femoral comum (AFC) e distal da artéria femoral superficial (AFS) e artéria femoral profunda (AFP) após a dilatação. Nota-se ligadura de ramos originados do aneurisma da AFP.

**Figura 4:** Resultado da revascularização, com interposição da prótese de Dacron® e anastomose proximal com artéria femoral comum (AFC) e distal com artéria femoral profunda (AFP), associado a reimplante da artéria femoral superficial (AFS) na prótese.

**Figura 5:** Peça cirúrgica. Morfologia do aneurisma e sua relação com a dimensão do cabo de bisturi número 7 e lâmina 11. No destaque: morfologia do aneurisma após abertura, observando-se trombos intraluminais.

**Figura 6:** Análise histopatológica. Coloração H&E: escala de x400. Artéria com lúmen dilatado, camada média degenerada e adventícia contendo inflamação crônica.

**Figura 7:** Análise histopatológica. Coloração H&E: escala de x400. Trombo fibrino-hemático agudo em processo de organização.

## DISCUSSÃO

Em revisão sistemática de artigos publicados em língua inglesa, foram encontrados 46 relatos de aneurisma verdadeiro isolado da AFP^6^. Em outra revisão no ano anterior, identificou uma série de pouco mais de 50 casos relatados^7^. Recentemente, aproximadamente 140 casos de aneurismas de AFP foram descritos na literatura^11^, mostrando que, ainda que crescente, são números que reforçam a raridade dessa entidade clínica.

Geralmente, os aneurismas de artérias femorais estão associados a aneurismas em outras topografias, como aorta e membros inferiores, e são bilaterais em 5% dos casos^1^^,^^4^^-^8. Em 50% dos casos, há associação com DAP no território fêmoro-poplíteo^4^. Os aneurismas falsos e pseudoaneurismas são mais comumente encontrados na literatura, principalmente os casos pós-traumáticos e iatrogênicos^6^^,^^12^. Considera-se que a baixa incidência se deva a contenção oferecida pelas estruturas musculares adjacentes, em especial o músculo adutor magno, envolvendo e protegendo a artéria^3^^,^^5^^-^8^,^^11^.

Devido à dificuldade do diagnóstico clínico, principalmente quando o aneurisma apresenta-se de pequeno tamanho, são comuns os casos assintomáticos ou com sintomas inespecíficos do membro inferior, como dor, edema e sintomas neurológicos^3^^-^7^,^^9^^,^^11^. O achado mais frequente é a presença de massa pulsátil com ou sem dor, porém podem apresentar-se como achados incidentais em exames complementares^3^^,^^6^. Há ainda relato de diagnóstico diferencial com hérnia inguinal encarcerada, principalmente em idosos, o que pode causar consequências desastrosas caso não seja diagnosticada antes da operação^2^.

O risco de complicações do aneurisma de AFP é elevado, frente à dificuldade no seu diagnóstico e, portanto, ao maior tempo sem tratamento da doença, o que pode levar o paciente a apresentar inicialmente trombose, embolização distal e ruptura^4^^,^^9^. O risco de rotura, a complicação mais comum, varia entre 15 e 55%, com associação direta do tamanho do aneurisma com o risco de rotura, sendo o diâmetro dos aneurismas da AFP geralmente maior que o da AFS^3^^,^^6^^,^^7^^,^^13^. A dificuldade de reconstrução devido a distorção da anatomia contribui para a elevada taxa de amputação quando os aneurismas evoluem com rotura^4^^,^^6^.

Na literatura, há diversas opções de abordagens cirúrgicas^11^, como ligadura e ressecção isolada do aneurisma^3^^,^^6^^-^8^,^^11^ ou associadas ou à revascularização^4^^,^^9^ com material sintético ou autólogo, além de procedimentos endovasculares por implante de stent recoberto^1^^,^^13^ e embolização, porém ainda não se dispõe de dados sobre a perviedade a longo prazo dos stents revestidos nessa topografia^1^. Podem ainda ser limitações para o tratamento endovascular: a extensão da doença para a AFC ou ainda a ausência de colo proximal de extensão adequada para o selamento proximal. Considera-se a ligadura segura, particularmente na presença de pulsos distais e de segmento femoro-poplíteo pérvio; no entanto, todo esforço deve ser feito para a revascularização quando o pulso distal está ausente^6^^,^^7^^,^^9^^,^^11^. A AFP desempenha importante papel na circulação colateral do membro inferior de paciente com DAP, com progressão ao longo dos anos^4^.

Para estabelecer o diagnóstico de certeza, o EDC, a ATC e a angiografia são extremamente úteis. A ATC de múltiplos detectores ou angiografia é especialmente recomendada para evidenciar a presença de outros aneurismas e lesões arteriais estenóticas ou oclusivas^3^^,^^7^. É necessário atentar-se também à anatomia e suas alterações, visto que há descrição de variações anatômicas nessa área que podem levar a desastres durante procedimentos, caso não tenham sido identificadas previamente^14^.

## CONCLUSÕES

No caso relatado, o paciente apresentava aneurisma de AFP associada a DAP e, portanto, havia necessidade de revascularização para manutenção da circulação colateral e perfusão adequada do membro inferior. Ofereceu-se ao paciente opção terapêutica cirúrgica efetiva e segura para o tratamento da doença, sem complicações pós-operatória a curto prazo. Dessa forma, deve-se considerar que a cirurgia convencional ocupe papel de destaque no tratamento dos aneurismas acometendo a AFP.
